# Frequency of cardiac arrhythmias in high- and low- yielding dairy cows 

**Published:** 2014

**Authors:** Afshin Jafari Dehkordi, Abdonnaser Nasser Mohebi, Masoumeh Heidari Soreshjani

**Affiliations:** 1 *Department of Clinical Science, Faculty of Veterinary Medicine, Shahrekord University, Shahrekord, Iran; *; 2 *DVM Student, Faculty of Veterinary Medicine, Shahrekord University, Shahrekord, Iran.*

**Keywords:** Arrhythmia, Blood serum, Cattle, Element, Milk production

## Abstract

Electrocardiography (ECG) may be used to recognize cardiac disorders. Levels of milk production may change the serum electrolytes which its imbalance has a role in cardiac arrhythmia. Fifty high yielding and fifty low yielding Holstein dairy cows were used in this study. Electrocardiography was recorded by base-apex lead and blood samples were collected from jugular vein for measurement of serum elements such as sodium, potassium, calcium, phosphorous, iron and magnesium. Cardiac dysrhythmias were detected more frequent in low yielding Holstein cows (62.00%) compared to high yielding Holstein cows (46.00%). The cardiac dysrhythmias that were observed in low yielding Holstein cows included sinus arrhythmia (34.70%), wandering pacemaker (22.45 %), bradycardia (18.37%), tachycardia (10.20%), atrial premature beat (2.04%), sinoatrial block (2.04%), atrial fibrillation (8.16%) and atrial tachycardia (2.04%). The cardiac dysrhythmias were observed in high yielding Holstein cows including, sinus arrhythmia (86.95%) and wandering pacemaker (13.05%). Also, notched P wave was observed to be 30% and 14% in high- and low- yielding Holstein cows respectively. The serum calcium concentration of low yielding Holstein cows was significantly lower than that of high yielding Holstein cows. There was not any detectable significant difference in other serum elements between high- and low- yielding Holstein cows. Based on the result of present study, could be concluded that low serum concentration of calcium results to more frequent dysrhythmias in low yielding Holstein cows.

## Introduction

In large animals, electrocardiography is confined to a simple base apex lead system to examine for conduction disturbances and arrhythmias, which are detected by measurement of the various waveforms and intervals in the electrocardiography (ECG) that represent depolarization and repolarization in the heart, and by observation of their absence or abnormality.^1^^-^^3^ 

The rate and rhythm of the heart, is influenced primarily by the integrity of the pacemaker, the conduction system and the myocardium, and also by the influence of the autonomic nervous system. Variation in the rate and rhythm can occur in normal animals due to strong or varying autonomic influence but can also be a reflection of primary myocardial disease. Other factors such as acid-base and electrolyte imbalance, potassium, sodium, magnesium and calcium, can influence rate and rhythm. Therefore, these factors must be taken into consideration in the assessment of apparent abnormalities detected on clinical examination of the cardiovascular system.^2^^,^^4^^-^^7^ 

The occurrence of conduction and myocardial disturbances is probably more common than generally recognized, because an electrocardiogram is usually only taken from animals in which there have been prior clinical indications of conduction abnormalities.^2^^,^^8^

A rapid increase of K^+^ level due to a preparation infusion in low K^+^ or in a K^+^ depleted animal or human that may result in a bradycardia or cardiac arrest.^9^^,^^10^ 

Potassium administration to an intact animal under light anesthesia results in an initial sinus tachycardia (at K^+^ levels of about 6.50 mEq L^-1^) followed by a gradual slowing of the sinus rate as the plasma K^+^ rises to a level of 7.50 to 8.00 mEq L^-1^. Sinoatrial block either of the Wenkebach or Mobitz II variety is seen both in the experimental and clinical "spontaneous" hyperkalemia. This is probably due to the fact that SA fibers are much more resistant to the depressing action of K^+^ than is the atrial myocardium.^   11^

Calcium has an effect on the TP (threshold potential), hence, hypercalcemia reduces (less negative) and hypo-calcemia increases (more negative) its magnitude, but does not significantly affect either the TRP (trans-membrane resting potential), shape, or amplitude of the AP (action potential).^   11 ^

Elevation of extracellular Mg^2+^ to 3-5 mmol L^-1^ depresses A-V conduction. This may be due to slowing of the upstroke velocity of phase 0, a mechanism similar to or identical with that induced by hyperkalemia.^9^^,^^11^^-^^13^ 

Iron and oxygen-derived free radicals are important in mediating post-ischemic cardiac injury probably because of the formation of hydroxyl radicals. Sullivan proposed that iron depletion protects against ischemic heart disease. An iron supplemented diet increased the degree of oxidative injury in ischemic rat hearts.^14^^-^^16^ 

Milk production may alter the concentration of some blood electrolytes in high yielding Holstein cows which may lead to changes in cardiac rhythm. Thus, the aim of this study was to evaluate the electrocardiographic features and levels of some blood serum elements in high- and low- yielding Holstein dairy cows.

## Materials and Methods

Fifty 4-year old high yielding Holstein dairy cows and fifty 4-year old low yielding Holstein dairy cows were used in this study (The animals were healthy without clinical signs and any organ abnormalities). All procedures were performed in a large dairy farm (Zagros Dairy farm, Shahrekord, Iran including 1700 animals). 

The average 305-day milk yield of high- and low- yielding Holstein dairy cows were 10600 and 6000 kg milk respectively with 3.50% fat and 3.25% protein (days in milk were 100 in both groups). Cows were fed according to their requirements for maintenance and milk production (NRC, 2001).^17^ The ration consisted of high quality roughages (maize silage and sugar beet pulp), soybean meal, concentrates (corn and barley) and mineral and vitamin supplement. 

Each animal was kept in a stock and given at least 10 min to be relaxed. A base apex bipolar lead was used for recording ECG. After spraying of the area with ethanol as a degreasing agent, the positive electrode of lead I (left arm) was placed to the skin of the left thorax at the fifth intercostal space, caudal to the olecranon and the negative electrode (right arm) was attached on the jugular furrow in the caudal third of the right neck. 

The earth electrode was attached to the skin of the left flank. The ECG was recorded during 3 to 5 min (animal in a relaxed state). The ECGs were obtained on a single channel machine (Model 110 Class I; Suzuken-Kenz, Nagoya, Japan) with the paper speed 25 mm sec^-1^ and calibration of 10 mm equal to 10 mV. All of the ECGs were examined by two of the authors independently, and finally both authors interpreted all dysrhythmias together. Blood samples were collected from jugular vein and delivered to laboratory. The serum was separated and stored at –20 ˚C until analysis of serum elements such as calcium, phosphorous, iron, magnesium, sodium and potassium.

The following step by step approach used for ECG interpretation:

1. Identify all the QRS complexes. Each QRS complex should be followed by a T wave, and the QT interval should be similar for all QRS configurations, unless there is a marked change in heart rate. Identify the remaining complexes. Are P waves, "F" (flutter) waves or "f" (fibrillation) waves present? Are there any artifacts?

2. Determine the atrial and ventricular rates. Are they identical? Is one too fast or too slow? This determines whether there is a tachycardia or bradycardia.

3. Are the P-P and R-R intervals regular? Determine whether an irregular rhythm has underlying regularity that is interrupted by irregular intervals or whether the rhythm is consistently irregular. Second-degree AV block and atrial and ventricular premature beats are arrhythmias with underlying regularity, whereas atrial fibrillation, sinus arrhythmia, and sinus arrest are truly irregular rhythms.

4. Are P waves present? If so, is there a P wave preceding every QRS complex? If not, there are premature depolarizations, escape beats, or atrial fibrillation. Are all P waves followed by QRS complexes? If not, second degree AV block may be present. Is the resultant P-R interval constant? If not, there may be a wandering pacemaker or first-degree AV block.

5. Are all P waves and QRS complexes identical or normal in contour? If not, this signifies more than one pacemaker, premature depolarizations, or escape beats.^1^^,^^2^

The blood serum components were determined at Clinical Pathology Laboratory, Department of Clinical Sciences, Shahrekord University. Serum total calcium (S-tCa) was determined by colourimetric method using commercial kit (Darman Kave Company, Isfahan, Iran). The content of inorganic phosphorus was determined photometrically using the commercial kit (Pars Azmoon Company, Tehran, Iran). The determination of magnesium levels in serum of cows was carried out using atomic absorption spectro-photometer (Model Unicam 939; Thermo Electron, Andover, USA). Concentrations of sodium and potassium in serum were estimated by flame photometer (Model PFP7; Jenway, Essex, UK) in 1:200 diluted samples using the procedures explained by Evans.^18^

The data were analyzed using SPSS (Version 14; SPSS Inc., Chicago, USA). To determine the significant difference between groups one way ANOVA was used. Probability of *p *< 0.05 were considered to be statically significant.

## Results

The serum elements of high- and low- yielding Holstein dairy cows are shown in Table 1. The types and number of cardiac dysrhythmias are given in Table 2. Cardiac dys-rhythmias were detected more in low yielding Holstein cows (62.00%) compared to high yielding ones (46.00%). 

**Table 1:** Mean ± SEM of serum elements in high and low yielding Holstein cows

**Parameters **	**High yielding ** **Holstein cows**	**Low yielding ** **Holstein cows**
**Calcium (mg dL** ^-1^ **)**	8.48 ± 0.18	7.75 ± 0.23*
**Phosphorous (mg dL** ^-1^ **)**	7.75 ± 0.51	7.73 ± 0.34
**Magnesium (mg dL** ^-1^ **)**	2.30 ± 0.19	1.70 ± 0.08
**Iron (µg dL** ^-1^ **) **	177.27 ± 17.13	150.39 ± 8.70
**Sodium (mEq L** ^-1^ **) **	141.12 ± 0.48	137.48 ± 0.70
**Potassium (mEq L** ^-1^ **)**	4.60 ± 0.05	4.14 ± 0.40

* Indicate significant difference between two groups, (*p *< 0.05).

**Table 2:** The frequency (%) of arrhythmias in high- and low- yielding Holstein cows

**Type of arrhythmias**	**High yielding ** **Holstein cows**	**Low yielding ** **Holstein cows**
**Sinus arrhythmia**	86.95	34.70
**Wandering pacemaker**	13.05	22.45
**Bradycardia**	0	18.37
**Sinus tachycardia **	0	10.20
**Atrial premature complex ** 0		2.04
**Atrial fibrillation **	0	8.16
**SA block **	0	2.04
**Atrial tachycardia**	0	2.04

**Fig. 1:**
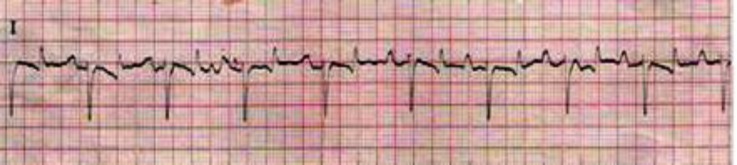
Sinus arrhythmia and wandering pacemaker in high yielding Holstein cow (Base apex lead, 25 mm sec^-1^ of 10 mm= 10 mV).

**Fig. 2:**
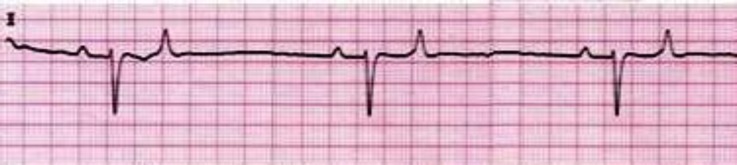
Bradycardia in low yielding Holstein cow (Base apex lead, 25 mm sec^-1^ of 10 mm= 10 mV).

**Fig. 3:**
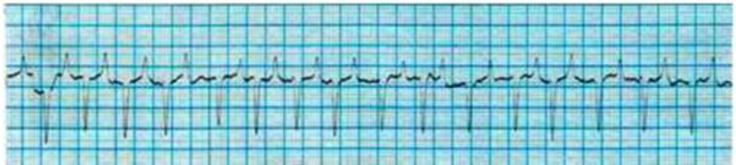
Atrial tachycardia in low yielding Holstein cow (Base apex lead, 25 mm sec^-1^ of 10 mm= 10 mV).

**Fig. 4:**
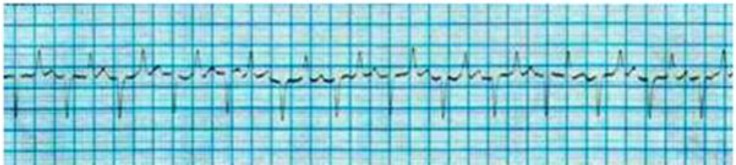
Sinus tachycardia in low yielding Holstein cow (Base apex lead, 25 mm sec^-1^ of 10 mm= 10 mV).

**Fig. 5:**
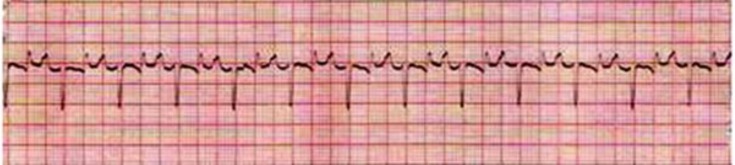
Notch P wave (Base apex lead, 25 mm sec^-1^ of 10 mm= 10 mV).

The cardiac dysrhythmias observed in low yielding Holstein cows were included sinus arrhythmia (34.70%), (Fig. 1); wandering pacemaker (22.45%), (Fig. 1); bradycardia (18.37%), (Fig. 2); tachycardia (10.20%), (Fig. 3); atrial premature beat (2.04%); sinoatrial block (2.04%); atrial fibrillation (8.16%) and atrial tachycardia (2.04%), (Fig. 4). The cardiac dysrhythmias observed in high yielding Holstein cows were included, sinus arrhythmia (86.95%) and wandering pacemaker (13.05%). Also, notched P wave was observed in high- and low- yielding Holstein cows, 30.00% and 14.00%, respectively (Fig. 5).

The serum calcium concentration of low yielding Holstein cows was significantly lower than that of high yielding Holstein cows. There was not any detectable significant difference of other serum elements between high- and low- yielding Holstein cows. 

## Discussion

Cardiac dysrhythmias or arrhythmias are defined as disturbances of impulse formation, disorders of impulse conduction, or both.^1^ In the present study, cardiac dysrhythmias were detected more in low yielding Holstein cows (62.00%) compared to high yielding Holstein cows (46.00%). Eight types of dysrhythmias, either alone or in combination with another type, were observed in the low yielding Holstein cows whereas two types of dysrhythmias detected in the high yielding ones (Table 2).

While a number of electrolytes play a role in the genesis of the transmembrane action potential (AP), the changes in the action potential most clearly related to arrhythmias are dependent to a large extent on K^+^. Potassium gradient is a major determinant of the magnitude of transmembrane resting potential (TRP), and secondarily the rate of rise (dV/dt) of phase 0, and consequently the speed of conduction. The cell membrane conductance for K^+^, or a decrease therein, is most likely the major determinant of spontaneous slow depolarization during phase 4. Thus, K^+^ has a pronounced effect on both conduction and automaticity. Furthermore, Ca^2+^ Mg^2+^, or Na^+^ affect the action potential and induce experimental arrhythmias at concentrations which are non-physiologic and frequently incompatible with life.^   11 ^ Also, there is evidence concerning the role of iron in atherosclerosis and ischemic heart disease.^14^^,^^16^

However, in this study there were not any detectable significant changes in serum magnesium, phosphorous, sodium, potassium and iron concentration between two groups of high- and low- yielding cows.

The serum calcium level in the high yielding Holstein cows (8.48 ± 0.18 mg dL^-1^) was significantly higher than low yielding Holstein cows (7.75± 0.23 mg dL^-1^) whereas, the calcium requirements in diets were formulated according to animal requirements in both groups. Therefore, there was not any calcium deficiency in the diet of both groups. Thus, it seems that calcium and phosphorous hemostasis is more active in high yielding Holstein cows than low yielding Holstein cows as a result of high milk production that results in high production of parathormone to maintain blood calcium to normal value. 

In this study, cardiac dysrhythmias were detected more in low yielding Holstein cows (62.00%) as compared to high yielding Holstein cows (46.00%) that may be caused by the lower serum calcium level in low yielding Holstein cows than high yielding Holstein cows. Calcium has an effect on the TP. Therefore, hypercalcaemia reduces (less negative) and hypocalcaemia increases (more negative) its magnitude, but does not significantly affect the TRP, shape, or amplitude of the AP. Hypocalcaemia reduces the TRP and increases the slope of phase 4 depolarization. In the intact experimental animal, marked elevation of the plasma Ca^2+^ may induce depression of intraventricular conduction, ventricular premature systoles, and fibrillation: In patients with extreme elevation of Ca^2+^, prolongation of P amplitude, or higher degrees of A-V block and prolongation of the QRS have been reported.^11^^,^^19^^,^ ^20^

Decrease in extracellular calcium concentration prolonged the duration and reduced amplitude of second changes of membrane action potential that appears on electrocardiogram in prolonged ST segment and increased QT segment.^   11 ^

Rezakhani and Sayari investigated the arrhythmia after intravenous injection of calcium in cows. They reported bradycardia, sinoatrial block, atrioventricular block, ventricular escape beat, and sinus tachycardia.^   2 1^

Littledike *et al.* believed that hypercalcemia causes hypertension and affects the vagus activity that leads to arrhythmia, because atropine reduce arrhythmia outcome calcium injection.^   1 9^

 Rezakhani *et al.* reported seven arrhythmias, single or with other arrhythmia, in healthy cows which sinus arrhythmia was most common in them.^   8 ^

In conclusion, this study showed that cardiac dysrhythmias were detected more in low yielding Holstein cows compared to that of high yielding Holstein dairy cows. Therefore, based on the result of the present study, could be concluded that low serum concentration of calcium results to more frequent dysrhythmias in low-yielding cows.
